# An Entropy-Based Approach to Measurement of Stock Market Depth

**DOI:** 10.3390/e23050568

**Published:** 2021-05-03

**Authors:** Joanna Olbryś, Krzysztof Ostrowski

**Affiliations:** Faculty of Computer Science, Bialystok University of Technology, 15-351 Bialystok, Poland; k.ostrowski@pb.edu.pl

**Keywords:** entropy, market microstructure, dimensions of market liquidity, market depth, high-frequency data, intra-day seasonality

## Abstract

The aim of this study is to investigate market depth as a stock market liquidity dimension. A new methodology for market depth measurement exactly based on Shannon information entropy for high-frequency data is introduced and utilized. The proposed entropy-based market depth indicator is supported by an algorithm inferring the initiator of a trade. This new indicator seems to be a promising liquidity measure. Both market entropy and market liquidity can be directly measured by the new indicator. The findings of empirical experiments for real-data with a time stamp rounded to the nearest second from the Warsaw Stock Exchange (WSE) confirm that the new proxy enables us to effectively compare market depth and liquidity for different equities. Robustness tests and statistical analyses are conducted. Furthermore, an intra-day seasonality assessment is provided. Results indicate that the entropy-based approach can be considered as an auspicious market depth and liquidity proxy with an intuitive base for both theoretical and empirical analyses in financial markets.

## 1. Introduction

The original idea of entropy dates back to the fundamental Shannon’s theory of communication and information [1]. Entropy was defined by Shannon as a measure of information, choice and uncertainty. The concept of entropy originates from thermodynamics, but it has been utilized in many research fields to characterize the complexity of a system and to investigate the information content of a probability distribution. Entropy is a general measure, and therefore, many definitions and applications of entropy have been proposed in the literature. Since the aim of this paper is to introduce and utilize a new entropy-based estimator of stock market depth as one of stock market liquidity dimensions, the brief literature review focuses on selected entropy-based applications in economics, finance, and management. Theoretical, empirical, and experimental aspects of entropy utilization are highlighted.

Firstly, there are quite many entropy-based applications in portfolio selection, asset pricing, and risk management, including the entropy optimization approach. Zhou et al. [2] presents a comprehensive review of applications of entropy in finance. Both primary and recent studies are included. For instance, the pioneering work of Philippatos and Wilson [3] proposed the mean-entropy concept in the efficient portfolio selection problem. The main contribution of this primary research lies in the conclusions that mean-entropy portfolios are consistent with the Markowitz and Sharpe models.

In light of the recently growing literature on the entropy-based applications, the topic concerning portfolio selection is very popular. Among others, Usta and Kantar [4] present a multi-objective method based on a mean-variance-skewness-entropy portfolio selection model to generate a well-diversified portfolio. Zhang et al. [5] deal with a multi-period portfolio selection problem with fuzzy returns. In this paper, the diversification degree of a portfolio is measured by the originally presented possibilistic entropy. Huang [6] proposes two types of credibility-based fuzzy mean-entropy models for fuzzy portfolio selection, and entropy is used as the measure of risk. Yu et al. [7] evaluate the performance of the portfolio models that are used to rebalance with short selling, considering transaction costs, minimizing portfolio risk, and utilizing entropy in modeling asset allocation. Zhou et al. [8] systematically explore the properties of six kinds of entropy-based risk measures, and develop and compare several portfolio models based on different risk measures. Yang and Qiu [9] extend the classical decision model under risk to a more general case. They propose an expected utility-entropy measure of risk and a decision-making model based on expected utility and entropy. Pele et al. [10] investigate relationship between the information entropy of the distribution of intraday returns, and intraday and daily proxies of financial market risk. They use Value-at-Risk and Expected Shortfall as risk measures for the EUR/JPY exchange rate. Gradojevic and Caric [11] concentrate on quantifying the behavioral aspects of systematic risk by utilizing a novel entropy-based approach. Their empirical results confirm the predictive usefulness of new entropy setting in stock market risk management.

In the mathematical finance literature, there are several papers dealing with entropy as an optimization criterion, especially in the context of asset and option pricing. For example, Fritelli [12] investigates the properties of the minimal entropy martingale measure, and shows that the minimization of relative entropy is equivalent to the maximization of expected exponential utility of wealth. Stutzer [13] proposes relative entropy minimization approach to derivation of a generalized Black-Scholes option pricing model. In their theoretical research concerning risk management, Geman et al. [14] use entropy maximization approach to recognize the uncertainty of asset distribution. Xu et al. [15] propose a continuous maximum entropy method to analyze the robust optimal portfolio selection problem in the case of the market with transaction costs and dividends. Brody and Hughston [16] introduce a new term structure calibration methodology based on maximization of entropy, and present some new models of interest rate. Gulko [17,18] applies the Entropy Pricing Theory to introduce new formulas for pricing European stock and bond options.

Another broad research field is information and entropy econometrics that directly or indirectly builds on the foundations of information theory and the principle of maximum entropy. Among other topics, Golan and Perloff [19] deeply investigate the generalized maximum entropy estimation method. Ullah [20] provides the uses of entropy and divergence measures for evaluating econometric approximations and inference. Kitamura and Stutzer [21] develop the relationship between entropic and linear projections in asset pricing estimation based on stochastic discount factor models. Maasoumi and Racine [22] examine the predictability of stock market returns by employing a new metric entropy measure and compare their results with a number of traditional measures. Bera and Park [23] use maximum entropy portfolio selection method in the optimal portfolio diversification problem, and their approach can be viewed as a shrinkage estimation of portfolio weights.

According to the literature, several studies propose entropy-based methods to investigate groups of stock markets in the world, especially in the context of various common features, relationships and interdependences between them. For instance, Billio et al. [24] use different entropy measures and new early warning indicator for banking crises to analyze the time evolution of systematic risk in Europe. They focus on the euro zone and analyze a total of 437 European financial institutions. Zhao et al. [25] propose copula entropy models to measure dependence in stock markets. The Authors provide an algorithm for the copula entropy approach to obtain the numerical results, and they approve the validity of the proposed method. Zunino et al. [26] introduce two quantifiers for a stock market inefficiency: the number of forbidden patterns and the normalized permutation entropy. The Authors analyze equity indexes and returns for 32 different stock exchanges. They point out that their empirical findings suggest that the proposed physical tools are helpful to discriminate the stage of stock market development.

Another promising strand of the literature concerns network entropy since Mantegna [27] first represented the financial market as a network. Financial markets are complex systems and can be represented as complex networks. Network entropy can be treated is a measure of information contained in the system [28,29].

It is worth noting that quite extensive studies consider the concepts of mutual information and transfer entropy. These tools enable us to investigate the information flow between time series and are especially useful in economic and financial applications [30,31,32,33,34,35,36,37,38].

The literature contains several theoretical, empirical, and experimental studies concerning entropy-based applications in market microstructure research. For instance, Liu et al. [39] use entropy-based measures to identify various types of trading behaviors. Albeit, the papers regarding dimensions of market liquidity are relatively scarce. For instance, McCauley [40] points out that interest in thermodynamic analogies in economics and finance is older than the idea of von Neumann to look for market entropy in liquidity. McCauley assumes that the definition of an asset’s liquidity is analogous to this of stock market depth. However, he concludes that real financial markets cannot behave thermodynamically because they are unstable.

Order imbalance has a significant influence on stock illiquidity, considerably more important even than volume. In the recent paper, Lu et al. [41] proposed an indicator called polarity to investigate trading imbalance in Chinese stock market. This indicator is based on high-frequency transaction data. However, the definition of polarity is very similar to this of order ratio, which is well known and broadly used in the literature as an indicator of stock market depth and market illiquidity (see e.g., [42,43] and the references therein). Therefore, the aforementioned paper was our research inspiration and motivation for taking and exploring the subject of an entropy-based approach to measurement of stock market depth as one of market liquidity dimensions [44].

The aim of this study is to introduce a new entropy-based market depth proxy that is exactly based on the definition of Shannon information entropy [1]. Our proposition substantially differs from the entropy-based indicator of trading imbalance presented in [39] because we employ the Lee and Ready [45] algorithm inferring the initiator of a trade to distinguish between so-called buyer- and seller-initiated trades.

The value-added of this research derives both from the new methodology and novel empirical findings. There are some advantages of the proposed indicator. Firstly, it can be treated as a new measure of stock market liquidity. The values of the entropy-based market depth are decimal fractions that vary between zero and the exactly defined maximal value equal to one. Therefore, the entropy-based market depth values calculated for different equities can be effectively compared to each other.

Moreover, based on the Shannon entropy definition, the entropy-based market depth indicator can be used to summarize the information content of a probability distribution, and it can be treated as a measure of stock market efficiency according to the Efficient Market Hypothesis (EMH). High values of entropy are related to randomness in the evolution of stock prices [26]. Higher values of market entropy inform about higher market efficiency, and are coupled with higher values of stock liquidity. Therefore, both market entropy and market liquidity can be directly measured by the proposed new indicator.

Empirical experiments on financial markets depend on data availability. Therefore, the real-data experiments and statistical analyses are conducted for high-frequency data with a time stamp rounded to the nearest second from the Warsaw Stock Exchange (WSE). Stability and robustness tests are conducted. Moreover, an intra-day seasonality assessment is provided to recognize intra-day hourly patterns in new entropy-based market depth indicator. Results indicate that this indicator can be considered as an auspicious market depth measure with an intuitive base for both theoretical and empirical analyses in financial markets. The proposed entropy-based indictor can be successfully utilized using intra-day data from other stock markets in the world, and the results could be interesting for investors.

The remainder of the study is organized as follows: Section 2 specifies the methodological background of measurement of market depth in the context of the broad topic concerning dimensions of stock market liquidity. Section 3 contains real-data description and presents the findings of some empirical experiments, statistical analyses, and robustness tests for high-frequency data. In Section 4, we discuss and conclude the results and propose several directions for further research.

## 2. Methods

### 2.1. Depth as One of Stock Market Liquidity Dimensions

Generally speaking, stock market liquidity is not a one-dimensional variable. The literature concerning the dimensions of market liquidity has continued to grow since Kyle [44] first distinguished between three dimensions: depth, tightness, and resiliency. Depth can be defined as the ability to buy or sell a certain amount of an asset without influence on the quoted price. In other words, the depth of market captures the relation between order flow and price changes. When demand (buy) and supply (sell) sides are quantitatively the same, the quoted price will not change (there is no impulse to price changes). The definition of tightness states that this is the ability to buy and to sell an asset at about the same price at the same time. One of definitions of market resiliency specifies that this is the ability to buy or to sell a certain amount of an asset with little influence on the quoted price. Theoretical and empirical findings of research on liquidity dimensions in several stock markets in the world are reported in [46,47,48,49,50,51,52].

It is pertinent to notice that the studies that explore depth, tightness, and resiliency as dimensions of stock market liquidity on the WSE are rather scarce. For instance, order imbalance as a measure of market depth is assessed in the papers [42,43,53]. Market tightness as the cost of turning around a position over a short period of time on the WSE is investigated in the works [42,43]. Moreover, two new methods for measurement of intraday stock market resiliency based on the Discrete Fourier Transform and Short-Time Fourier Transform approaches are introduced and utilized for high-frequency data from the WSE in the recent papers [54,55].

### 2.2. Measuring Stock Market Depth

Related literature proposes various proxies of stock market depth, and a comprehensive review of them is presented e.g., in [42]. In general, the measures of order imbalance are the most frequently used.

To introduce a new entropy-based method, firstly we propose a supporting modified version of the Order Ratio (OR) indicator as a refined proxy of market depth which accurately captures market order imbalance. It is defined by the following Equation (1):

(1) $$OR=\frac{\left|CTV_{b}-CTV_{s}\right|}{CTV_{b}+CTV_{s}},$$

where OR∈[0, 1] and the sums $CTV_{b}≔\sum_{i=1}^{m}Vbuy_{i}$, $CTV_{s}≔\sum_{j=1}^{k}Vsell_{j}$ denote the cumulated trading volume related to transactions classified as buyer- or seller-initiated trades, respectively. The modification lies in the denominator $\sum_{n=1}^{m+k}V_{n}=CTV_{b}+CTV_{s}$ which denotes the cumulated trading volume for all classified transactions within a particular period of time (in the frequently used version of the OR indicator, the denominator includes the cumulated trading volume for all transactions within an investigated period of time).

The OR indicator can be calculated within various time intervals, for example in 30-min, hourly or daily manner because the Formula (1) is the general one. The order ratio informs about imbalance in the market since it rises when the difference in the numerator rises, and therefore it measures illiquidity. High values of the order ratio indicate low market depth and low liquidity. Conversely, small values of this indicator denote high market depth and high liquidity. According to definition (1), the order ratio value is non-negative and it is equal to zero when cumulated trading volumes related to transactions classified as buyer- or seller-initiated trades are equal within a particular time interval. The order ratio value given by Equation (1) is not defined for the following two cases: (1) when all transactions within an analyzed time period are unclassified, and (2) an analyzed time period is a zero-volume period, which means the total lack of transactions. In such cases, the total trading volume in the denominator is equal to zero. The OR value is equal to one when all transactions within an analyzed time period are classified in the same manner (i.e., as only buyer- or only seller-initiated trades).

In the next step, the definitions of cumulated trading volumes related to transactions classified as buyer- or seller-initiated trades are used to define the probabilities and the entropy-based proxy of stock market depth. In light of the recently growing literature, entropy is a widely accepted measure of a generally understood diversity and disorder. In this context, an entropy-based indicator could represent the unevenness of buying and selling in trading decisions on a stock market [41]. Shannon [1] proves that quantities of the form $H=-K\cdot \sum_{i=1}^{n}p_{i}\cdot \log\left(p_{i}\right)$, where K is a positive constant that amounts to a choice of a unit of measure, play a central role in information theory as measures of information, choice, and uncertainty. Exactly based on the definition of Shannon information entropy [1] (p. 394) we propose the following new Entropy-based Market Depth (EMD) indicator given by Equation (2):

(2) $$EMD=\frac{-1}{\log(2)}\left(P_{buy}\cdot \log\left(P_{buy}\right)+P_{sell}\cdot \log\left(P_{sell}\right)\right),$$

where:

(3) $$P_{buy}=\frac{CTV_{b}}{CTV_{b}+CTV_{s}}\in \left[0,\;1\right],$$

(4) $$P_{sell}=1-P_{buy}=\frac{CTV_{s}}{CTV_{b}+CTV_{s}}\in \left[0,\;1\right].$$

According to the Shannon definition, the EMD indicator (2) measures the entropy in the case of two possibilities with probabilities defined by Equations (3) and (4). It is scaled to obtain the EMD values that belong to the [0;1] interval (without the normalization, the maximal EMD value is equal to log(2)≈0.301, and it is obvious based on the properties of the Shannon information entropy [1], p. 394). According to Equation (2), the EMD value is non-negative, and it is defined as equal to zero in the following two cases:

(1) If $CTV_{b}=0\Leftrightarrow P_{buy}=0\Leftrightarrow P_{sell}=1\Leftrightarrow \log\left(P_{sell}\right)=0\Leftrightarrow EMD=0$;
(2) If $CTV_{s}=0\Leftrightarrow P_{sell}=0\Leftrightarrow P_{buy}=1\Leftrightarrow \log\left(P_{buy}\right)=0\Leftrightarrow EMD=0$.

The EMD value given by Equation (2) is not defined for the following two cases: (1) when all transactions within an analyzed time period are unclassified, and (2) an analyzed time period is a zero-volume period, which means the total lack of transactions. In such cases, the total trading volume in the denominator in Equations (3) and (4) is equal to zero. Appendix A contains further justification for the EMD indicator in the context of the Shannon information entropy definition.

To calculate both the OR (1) and EMD (2) indicators using intraday data it is essential to recognize the side initiating a transaction. Although the WSE is a pure order-driven market with an electronic order book, information of the order book database is not publicly available. Thus, the side initiating a trade cannot be directly identified from a raw data set. Therefore, the Lee and Ready (LR) [43] algorithm inferring the initiator of a trade is used to distinguish between so-called buyer- and seller-initiated trades. Although several trade-side classification rules have been proposed in the literature, Olbryś and Mursztyn [56] confirm that the LR algorithm performs better than other procedures on the Polish stock market. For details about the LR algorithm see Table A1, in Appendix B.

As the EMD is a new both market depth and market liquidity indicator which is indirectly connected (via the probabilities defined by Equations (3) and (4)) with the supporting modified version of the OR proxy, it would be useful and informative to compare the OR and EMD values. Table 1 presents simple illustrative examples of calculations of both indicators for four selected cases within the same time period. Example 1 shows that the minimal OR value equal to zero is coupled with the maximal EMD value equal to one. In general, as the EMD values are decimal fractions that belong to the [0;1] interval, the EMD values calculated for different equities can be easily compared to each other. Furthermore, Examples 2–3 illustrate that increasing values of the OR are coupled with decreasing values of the EMD indicator, and vice versa. Example 4 shows that the maximal OR value equal to one is coupled with the minimal EMD value equal to zero. It means that, on an intuitive base, the maximal value of trading imbalance indicates the lack of liquidity.

**Figure 1:**
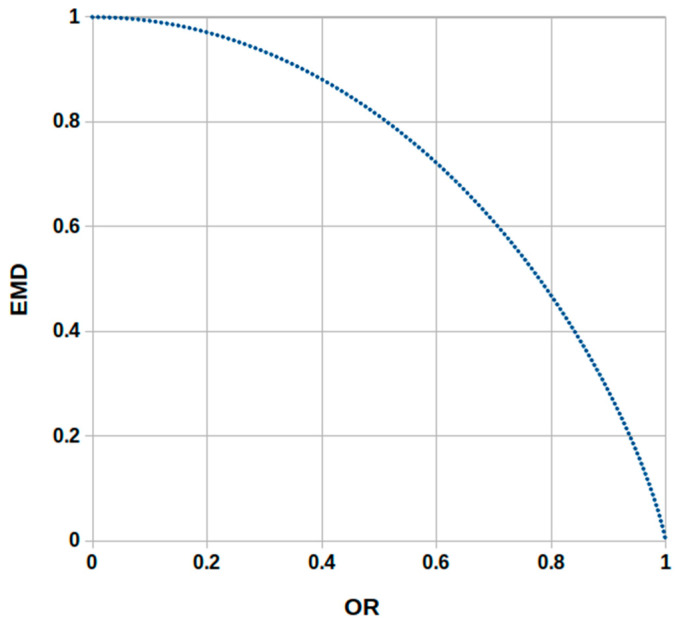
The relationship between the Entropy-based Market Depth (EMD) and Order Ratio (OR) indicators.

Figure 1 depicts the relationship between OR and EMD indicators. To sum up, the examples presented in Table 1 and Figure 1 show that low Order Ratio values are accompanied by high values of the Entropy-based Market Depth indicator. Otherwise, high ORs are accompanied by low values of the EMD indicator. This evidence is consistent with overall relations between these two depth estimates.

Table 2 briefly summarizes basic relationships between the indicators (1) and (2), market depth, market liquidity, and market entropy. As one can observe, the OR proxy is only a measure of depth and illiquidity, while the EMD can be treated as a measure of market depth, market liquidity and market entropy, which is the advantage of this new indicator.

## 3. Empirical Experiments for High-Frequency Intraday Data

As empirical experiments on financial markets depend on data availability, this section is devoted to the comparative and comprehensive investigation of the OR and EMD indicators on the Polish stock market. It presents findings of several empirical experiments and statistical analyses for high-frequency data from the Warsaw Stock Exchange. The database is large. It contains 21,010,718 records in total (see Table 3). Therefore, all computations were performed using a customized program (language C++, system: Linux, processor 3.6 GHz, RAM 4 GB). 

### 3.1. Real-Data Description

The sample contains high-frequency data for 20 WSE-listed companies with the largest market capitalization (MV) at the end of 2016. Tick-by-tick transaction data is not publicly available for the WSE. Thus, in this research transaction prices and volume records with a time stamp rounded to the nearest second, for each security over one unit of time are used. The data comes from the Bank for Environmental Protection (BOS) brokerage house (available at http://bossa.pl; accessed date 5 January 2017). All stocks included in the database have been incessantly listed on the WSE through the whole sample period. This study is the continuation and extension of the research on dimensions of market liquidity on the WSE presented in the papers [54,55], and therefore the database is the same.

The sample period ranges from 2 January 2005 to 30 December 2016 (3005 trading days). To verify the robustness of the empirical findings, the calculations are provided both for the whole sample and over three consecutive sub-samples of equal length (436 trading days) [54]:

(1) The pre-crisis sub-period from 6 September 2005 to 31 May 2007 (S1);
(2) The crisis sub-period on the WSE from 1 June 2007 to 27 February 2009 (S2);
(3) The post-crisis sub-period from 2 March 2009 to 19 November 2010 (S3).

The crisis sub-period on the WSE connected to the 2007–2009 Global Financial Crisis (GFC) period was formally defined based on the paper [57], in which the statistical method for the quantitative identification of market states is used.

### 3.2. Estimation Results of the Order Ratio and Entropy-Based Market Depth

This subsection includes brief information on the group of 20 WSE-traded companies that are analyzed in this research (Table 3). The companies are labeled by ticker symbols and presented in decreasing order of the market value (MV) at the end of 2016. Table 3 reports the numbers of records in the database for each stock and the averaged daily values of the OR and EMD indicators. Standard deviations are given in parentheses. The evidence is that for the most liquid equities with the largest numbers of records in the database (namely PKN, PKO, PEO, KGH, OPL) the averaged daily values of the OR and EMD indicators and standard deviations of these values are stable in time. The findings confirm high market depth and high liquidity of these stocks as the averaged EMD proxy is approximately equal to one and accompanied by very low standard deviations. What is important, the experimental results reported in Table 3 show that the precise ranges of OR [0.17; 0.68] and EMD [0.47; 0.98] are equally broad (0.51 for OR and 0.51 for EMD).

Table 4 reports Pearson correlation coefficients calculated for series of daily market depth estimators given by Equations (1) and (2), for each asset separately. This table presents the results for the whole sample (WS) and three sub-samples S1, S2, S3. All correlations are significantly negative and their absolute values are very high. This evidence confirms that the information content of both market depth proxies is the same, while the main advantage of the EMD indicator is that it measures liquidity (not illiquidity, like the OR estimate). This evidence is consistent with the relationship presented in Figure 1.

### 3.3. Robustness Tests of Entropy-Based Market Depth

Various robustness analyses are standard procedures for testing stability of stock market characteristics, especially in the context of crises periods, e.g., [24,41,54,55]. The existing studies indicate that the empirical results could be diverse and economic interpretations are needed in such cases. The Entropy-based Market Depth (EMD) indicator (2) is proposed as a new estimator of a stock market depth and market liquidity. Therefore, the stability of estimation results by time periods could be assessed. To address this issue, the robustness tests over the whole sample period and three sub-periods are provided. The goal is to investigate whether the mean results of stock depth and liquidity approximated by EMD within the analyzed periods (reported in Table 3) significantly differ between each other. The following two-tailed hypothesis is tested:

(5) $$\begin{array}{c} H_{0}:\mu_{1}=\mu_{2}\\ H_{1}:\mu_{1}\neq \mu_{2} \end{array},$$

where $\mu_{1},\mu_{2}$ are the expected values of depth for each equity within the compared periods, and the null hypothesis assumes that two expected values are equal.

To verify the hypotheses, the Z-statistic for independent large sample means is used:

(6) $$Z=\frac{\bar{x_{1}}-\bar{x_{2}}}{\sqrt{\frac{s_{1}^{2}}{n_{1}}+\frac{s_{2}^{2}}{n_{2}}}},$$

where $\bar{x_{1}},\;\bar{x_{2}}$ are sample means, $s_{1}^{2},s_{2}^{2}$ are sample variances, and $n_{1},n_{2}$ denote a sample size, respectively. The numbers $n_{1},n_{2}$ of trading days for each stock within each period are reported in Table A2, Appendix C. The average daily values of the EMD and standard deviations of these values are documented in Table 3. To address the multiple testing problem, the Bonferroni correction is used, and therefore the significance level is equal to $\bar{\alpha }=0.0025$. The critical value of Z-statistic (6) at 0,25% significance level is equal to 3.03 for each test (we thank an anonymous referee for this suggestion).

Six pairs of periods are investigated, i.e., WS/S1, WS/S2, WS/S3, S2/S1, S2/S3, and S1/S3. Summarized findings for the whole group of companies are presented in Table 5 and they require some comments and economic interpretations. The hypothesis $H_{0}$ indicates that the average EMD values are stable in time within compared periods. One can observe that for the companies PKN, OPL, ZWC, CAR, STP there are no reason to reject $H_{0}$ for all six cases, for the KGH—for five cases, and for KTY—for four cases. However, for the remaining equities the results are more diverse. After deep investigation of the obtained results we can assert that there are three main reasons of this phenomenon.

Firstly, although Table 3 documents that for the most liquid equities the averaged daily values of the EMD indicator are high and approximately the same, the values of standard deviation and the significantly diverse number of trading days within the analyzed periods (reported in Table A2, Appendix C) leads to rejection of the null hypothesis (5) for some isolated cases (for instance, for PKO and PEO).

Moreover, the results depend on the pair of the sub-periods. It is important to remind that the pre-crisis (S1), crisis (S2), and post-crisis (S3) periods on the WSE are investigated, and the crisis sub-period on the WSE is connected to the 2007–2009 Global Financial Crisis (GFC). Therefore, the findings inform whether the mean results of market depth and liquidity during the GFC period on the WSE significantly differ compared to the other periods. One can observe that in the case of the pairs: S2/S1 (crisis/pre-crisis), and S2/S3 (crisis/post-crisis), the hypothesis $H_{0}$ is outweighed by the hypothesis $H_{1}$ in 9 out of 20 and 5 out of 20 cases, respectively. Moreover, for the pair WS/S2 (whole sample/crisis) the number of $H_{1}$ is equal to 10. Therefore, we can conclude that the visible influence of the GFC on market depth and liquidity was present for the following equities, including five banks: BZW, ING, MBK, LPP, BHW, MIL, SNS, BDX, GTC, ORB, ECH. In general, market depth and liquidity significantly differed during the crisis sub-period on the WSE for several analyzed companies, but not for all of them. The EMD values for the most liquid companies were much more stable. This evidence is consistent with the studies that have utilized other liquidity proxies to assess stock market liquidity dimensions on the WSE during the GFC (e.g., [42,43]).

Furthermore, the whole sample period (WS) is long (12 years), and it includes the years of substantial changes in market liquidity. The WSE was a medium-size emerging stock market during this period. Especially, the level of liquidity within the pre-crisis period (S1) was lower compared to other periods for several companies, e.g., ING, MBK, LPP, BDX, ECH (see Table 3), while the level of liquidity during the post-crisis period (S3) was higher for many stocks. As a consequence, the hypothesis $H_{0}$ is outweighed by the hypothesis $H_{1}$ in 11 out of 20 (for the pair WS/S1) and 13 out of 20 cases (for the pair S1/S3). The total number of $H_{1}$ is equal to 57 out of 120. In conclusion, the results reported in Table 5 are not homogenous but they can be explained based on the WSE liquidity behavior within the whole sample period and remaining sub-periods.

### 3.4. Intra-Day Seasonality in Entropy-Based Market Depth

The aim of this subsection is to assess intra-day seasonality and recognize intra-day hourly patterns in the EMD indicator of market depth. According to the literature, there are some possible shapes of intra-day patterns in various stock market characteristics such as volumes, depths, spreads, returns, transaction costs, order flows, market resiliency, etc. (see e.g., [55,58,59,60,61,62,63,64,65,66,67,68] and the references therein). Goodhart and O’Hara [58] emphasize that a fundamental property of high-frequency data is that observations can occur at varying time intervals. Therefore, trades are not equally spaced over the day, which may result in intra-day ‘seasonal’ patterns in stock market activity. Empirical investigation and visualization of these patterns may be a useful tool for decision-making process and can help an investor to state how particular characteristics vary over a session. Some shapes of intra-day patterns in stock market are possible but it is not surprising that perfectly shaped visual patterns rarely appear. There are several attributes that help to differentiate the most important shapes such as: M-similar, U-similar, W-similar, inverted-U, J-similar, and inverted-J patterns [55].

To explore intra-day patterns in the EMD indicator, the average hourly values of this indicator are calculated for each equity within the whole sample period (WS) and three sub-periods (S1, S2, S3). The WSE is an order-driven market with an electronic order book. Therefore, liquidity is provided only by limit orders submitted by investors and there are no market makers who support liquidity. Table 6 presents short market trading schedule on the WSE and the notation concerning the trading hours (*H*_1_–*H*_8_).

**Figure 2:**
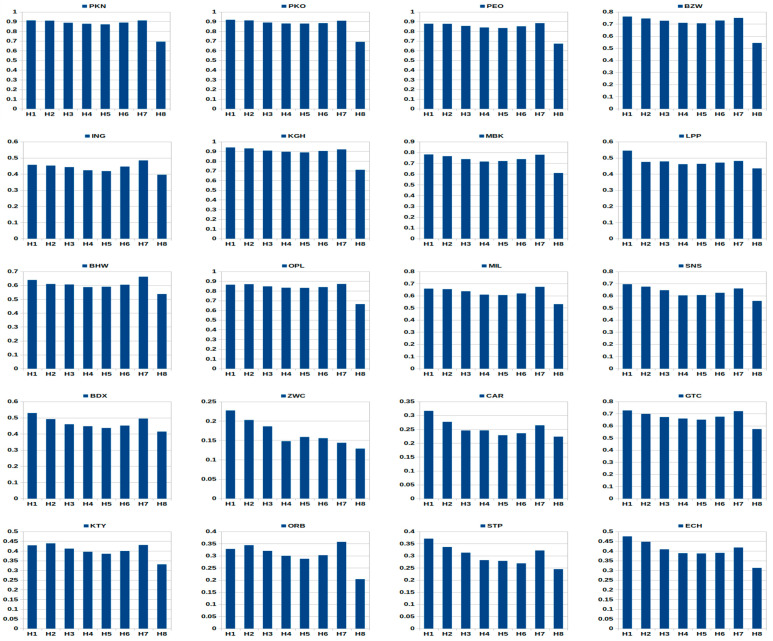
Intra-day hourly patterns of the EMD indicator within the whole sample period (WS) January 2005–December 2016 for the group of 20 WSE–listed equities. Notation as in Table 3 and Table 6.

**Figure A2:**
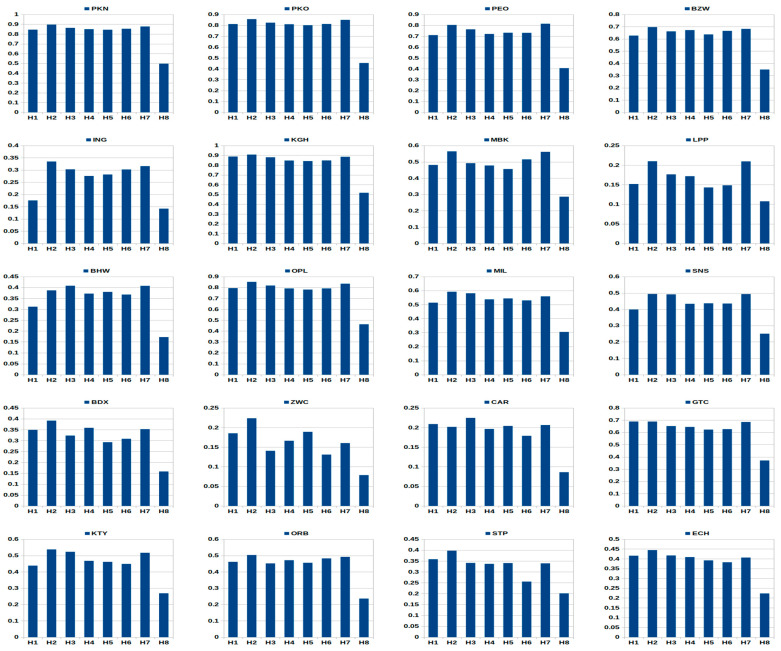
Intra-day hourly patterns of the EMD indicator within the pre-crisis period (S1) for the whole group of 20 WSE–listed stocks. Notation as in Table 3 and Table 6.

**Figure A3:**
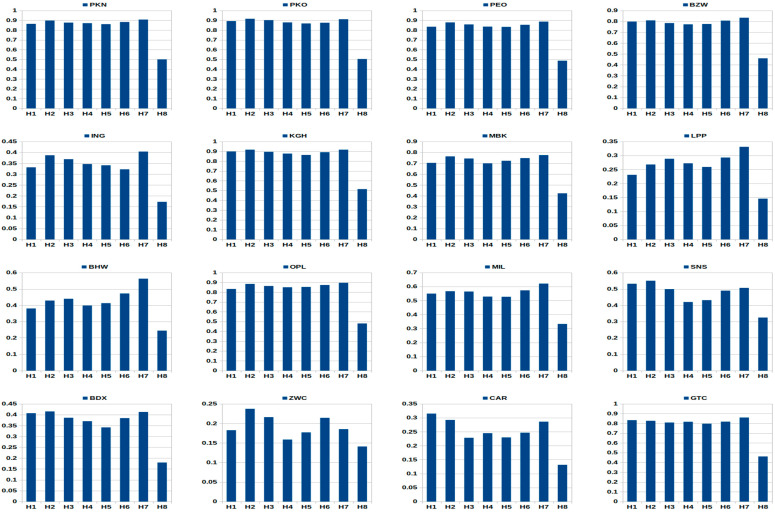

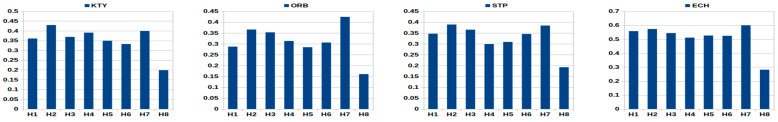
Intra-day hourly patterns of the EMD indicator within the crisis period (S2) for the whole group of 20 WSE–listed stocks. Notation as in Table 3 and Table 6.

**Figure A4:**
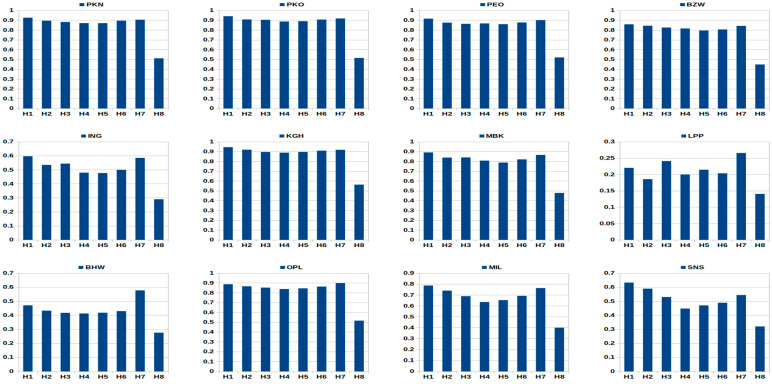

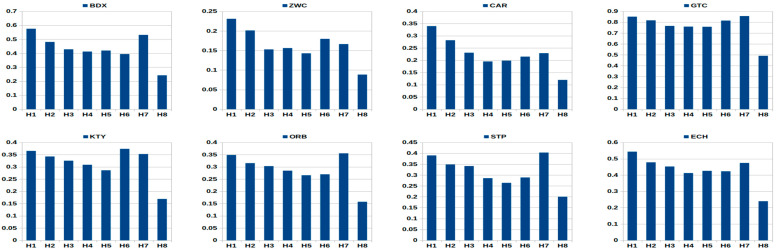
Intra-day hourly patterns of the EMD indicator within the post-crisis period (S3) for the whole group of 20 WSE–listed stocks. Notation as in Table 3 and Table 6.

Figure 2 illustrates hourly patterns in the EMD values within the whole sample period. The EMD intra-day behavior during remaining periods is presented in Figure A2, Figure A3 and Figure A4, Appendix D. Table 7 reports summarized findings of hourly patterns in the EMD indicator for the whole group of 20 WSE–listed equities investigated in this research. The trading hours *H*_1_–*H*_8_ based on Table 6.

It is important to notice that the results are homogenous. Except for isolated cases (e.g., LPP, ZWC), the M-similar and U-similar (with a decrease during the last hour H8) patterns dominate for the vast majority of stocks.

The M-shaped pattern depicts lower EMD values during the beginning and the ending of a session with the highest values slightly after the beginning and before the end. It is also marked by distinctively low value in the middle of a session.

The U-shaped pattern means that the value of the EMD decreases after the first hour. It then stays more or less constant, and increases during the last hour. In this context, the evidence concerning the U-similar pattern with a visible increase within the hour H7 and a pronounced decrease during the last hour H8 requires some explanations. It seems that this pattern is common for the most equities on the WSE. After deep investigation of the obtained empirical findings we can assert that the main reason of this phenomenon lies in the trade side classification results. Based on the Lee-Ready procedure presented in Appendix B (Table A1), two possible cases dominate within the last hour H8 on the WSE:

(1) The transactions are classified in the same manner (i.e., as only buyer- or only seller-initiated trades), which leads to EMD = 0 based on definition (2), and consequently decreases the average hourly value of the EMD. It’s common especially for less liquid companies with a small number of transactions in H8,
(2) The transactions classified as buyer- or seller-initiated trades dominate, which leads to small EMD values approx equal to 0 (see Table 1), and as a consequence decreases the average hourly value of the EMD.

Based on the summarized findings presented in Table 7 one can observe that, in general, the M-similar pattern dominates within the sub-periods S1 (pre-crisis) and S2 (crisis), while the U-similar pattern appears for vast majority of equities during the whole sample period and the sub-period S3 (post-crisis). In our opinion, the main reason of this phenomenon can be a higher level of market liquidity on the WSE after the GFC period.

It is worthwhile to emphasize that our results concerning intra-day behavior of the EMD indictor as a measure of liquidity are consistent with the literature. For instance, Jain and Joh [60] study joint characteristics of hourly common stock trading volume and returns and they find the U-shaped pattern in volume over the trading day on the New York Stock Exchange (NYSE). They emphasize that average volume as a liquidity proxy reveals significant hour of the day effect. McInish and Wood [62] show that number of shares traded as a liquidity estimate has a U-shaped intra-day pattern for all stocks listed on the Toronto Stock Exchange. Vo [64] also assess the intra-day behavior of market activity on the Canadian stock exchange in Toronto. The results confirm that spread follows U-shaped pattern, while volume is low at the open, stable during the day, and increases at the close. Ahn and Cheung [67] investigate the Stock Exchange of Hong Kong which is a pure electronic order-driven market without market makers.

The authors find the U-shaped patterns in spread and trading volume. As for the Polish stock market, Olbryś and Oleszczak [68] conduct empirical experiments for real-data from the WSE and they document that intra-day trading volume reveals U-similar or M-similar hourly patterns in the case of all investigated equities and for all analyzed periods.

## 4. Discussion and Conclusions

Concept of market depth focuses on the volume which can be observed at the current price level [49]. From investors’ and stock market analysts’ point o view, market depth is crucial because it can be treated as quantity dimension of market liquidity [51]. Harris [69] points out that the topic concerning dimensions of liquidity is especially interesting for practitioners as they often think about liquidity quite intuitively. Thinking about liquidity, investors usually think about trading quickly, trading large size, or trading at low costs.

According to the literature related to the microstructure of markets, several proxies of market depth are proposed: (1) depth as a number of units offered at the ask price plus a number of units at the bid price (e.g., [46,51,52]), (2) dollar depth calculated in currency terms (e.g., [70]), (3) an average depth of the ask and the bid (e.g., [71]), (4) an average dollar depth measured in currency terms (e.g., [71]), (5) various versions of order ratio as a proxy of realized market depth (e.g., [43,49,51,52,53]). The vast majority of these depth proxies require information about ask and bid prices.

However, although the WSE is a pure order-driven market with an electronic order book, information about ask and bid prices is not publicly available. Therefore, the side initiating a transaction cannot be directly identified from a data set. This problem concerns many emerging markets in the world, and a procedure inferring the initiator of a trade is needed in such cases.

Taking the above into consideration, this research contributes to the existing literature regarding dimensions of market liquidity by introducing and utilizing a new methodology for estimation of market depth and liquidity with the EMD indicator based on the Shannon entropy and supported by an algorithm inferring the initiator of a trade. The advantage of the EMD is that it measures liquidity, and the min and max values are in accordance with an investor’s intuition, i.e., EMD = 0 in the case of total illiquidity and EMD = 1 in the case of total liquidity. Hence, depth and liquidity calculated using the EMD for different stocks can be easily interpret and compared to each other. Moreover, the EMD can be treated as a measure of both market liquidity and market entropy. This is the advantage of this new indicator because higher values of entropy inform about higher market efficiency (in the sense of the EMH), and are coupled with higher values of stock liquidity.

Furthermore, intra-day behavior of the EMD indicator has been assessed and empirical findings concerning intra-day seasonal patterns in the EMD are homogenous and consistent with the existing studies on other liquidity proxies.

It is well documented in the literature that market depth varies with spread, volume, transactions, and volatility (see e.g., [49,51,70]). Therefore, one possible direction for future study could be an extensive econometric analysis of relationships between various stock market characteristics using the new EMD indicator as market depth proxy. Subject to data availability provision, the proposed entropy-based indictor could be utilized using high-frequency data from other stock markets in the world, and the results might be interesting for practitioners.

Another promising direction for further research might be to perform a theoretical analysis of the new entropy-based indictor from the perspective of the properties of extropy [72]. As the entropy and the extropy of a binary distribution are identical, the EMD indicator can be regarded also as an extropy measure (we would like to thank an anonymous referee for this valuable suggestion.).

## Figures and Tables

**Table 1:** Simple illustrative examples of the OR and EMD values for four selected cases and the same time period.

Possibilities	Probabilities	OR Indicator	EMD Indicator
Example 1—min OR and max EMD			
$CTV_{b}+CTV_{s}=1000$	$P_{buy}=\frac{500}{1000}=0.5$	$OR=\frac{\left|500-500\right|}{1000}=0$	$EMD=\frac{-1}{\log(2)}\left(2\cdot 0.5\cdot \log(0.5\right))=1$
$CTV_{b}=500$	$P_{sell}=\frac{500}{1000}=0.5$		
$CTV_{s}=500$			
Example 2			
$CTV_{b}+CTV_{s}=1000$	$P_{buy}=\frac{600}{1000}=0.6$	$OR=\frac{\left|600-400\right|}{1000}=0.2$	$EMD=\frac{-1}{\log(2)}\left(0.6\cdot \log(0.6)+0.4\cdot \log(0.4)\right)\approx 0.971$
$CTV_{b}=600$	$P_{sell}=\frac{400}{1000}=0.4$		
$CTV_{s}=400$			
Example 3			
$CTV_{b}+CTV_{s}=1000$	$P_{buy}=\frac{900}{1000}=0.9$	$OR=\frac{\left|900-100\right|}{1000}=0.8$	$EMD=\frac{-1}{\log(2)}\left(0.9\cdot \log(0.9)+0.1\cdot \log(0.1)\right)\approx 0.469$
$CTV_{b}=900$	$P_{sell}=\frac{100}{1000}=0.1$		
$CTV_{s}=100$			
Example 4—max OR and min EMD			
$CTV_{b}+CTV_{s}=1000$	$P_{buy}=\frac{1000}{1000}=1$	$OR=\frac{\left|1000-0\right|}{1000}=1$	$EMD=\frac{-1}{\log(2)}\left(1\cdot \log1+0\right)=0$
$CTV_{b}=1000$	$P_{sell}=\frac{0}{1000}=0$		
$CTV_{s}=0$			

**Table 2:** Overall relationships between two market depth indicators, market depth, market liquidity, and market entropy.

Indicator	Market Depth	Market Liquidity	Market Entropy
High order ratio (OR)	Low market depth	Low liquidity	–
Low order ratio (OR)	High market depth	High liquidity	–
High Entropy-based Market Depth (EMD)	High market depth	High liquidity	High entropy
Low Entropy-based Market Depth (EMD)	Low market depth	Low liquidity	Low entropy

**Table 3:** The averaged daily values of the Order Ratio (OR) and Entropy-based Market Depth (EMD) indicators within the whole sample period and three sub–periods for the group of 20 WSE–listed companies.

	Company	MV PLN m	No. of Records in the Database	OR indicator				EMD Indicator			
				WS	S1	S2	S3	WS	S1	S2	S3
1	PKN	36483.58	2739243	0.19 (0.13)	0.19 (0.13)	0.20 (0.14)	0.21 (0.13)	0.96 (0.05)	0.96 (0.05)	0.96 (0.05)	0.96 (0.05)
2	PKO	35175.00	3725299	0.19 (0.14)	0.24 (0.16)	0.21 (0.14)	0.20 (0.14)	0.96 (0.06)	0.94 (0.08)	0.95 (0.06)	0.96 (0.06)
3	PEO	33018.73	2210764	0.21 (0.16)	0.25 (0.19)	0.21 (0.15)	0.21 (0.15)	0.95 (0.08)	0.93 (0.11)	0.95 (0.06)	0.95 (0.06)
4	BZW	31358.11	996852	0.32 (0.25)	0.32 (0.23)	0.25 (0.18)	0.27 (0.19)	0.86 (0.21)	0.88 (0.17)	0.93 (0.10)	0.92 (0.10)
5	ING	20998.14	191091	0.48 (0.30)	0.60 (0.31)	0.55 (0.30)	0.44 (0.27)	0.73 (0.28)	0.60 (0.33)	0.67 (0.30)	0.78 (0.24)
6	KGH	18496.00	4582816	0.17 (0.13)	0.17 (0.12)	0.19 (0.14)	0.19 (0.13)	0.98 (0.05)	0.97 (0.05)	0.96 (0.05)	0.96 (0.05)
7	MBK	14174.12	930982	0.29 (0.21)	0.40 (0.27)	0.28 (0.21)	0.25 (0.17)	0.90 (0.14)	0.81 (0.22)	0.90 (0.13)	0.93 (0.08)
8	LPP	10412.23	275452	0.50 (0.33)	0.68 (0.32)	0.56 (0.31)	0.62 (0.33)	0.68 (0.35)	0.47 (0.38)	0.64 (0.33)	0.56 (0.36)
9	BHW	9981.09	498427	0.38 (0.27)	0.49 (0.30)	0.48 (0.29)	0.50 (0.29)	0.83 (0.23)	0.72 (0.29)	0.74 (0.27)	0.72 (0.27)
10	OPL	7231.09	2055914	0.22 (0.16)	0.20 (0.15)	0.20 (0.15)	0.22 (0.17)	0.95 (0.08)	0.95 (0.07)	0.95 (0.06)	0.94 (0.08)
11	MIL	6296.08	547539	0.34 (0.24)	0.40 (0.27)	0.39 (0.26)	0.30 (0.21)	0.86 (0.18)	0.81 (0.22)	0.82 (0.20)	0.90 (0.13)
12	SNS	6034.02	678481	0.32 (0.23)	0.43 (0.25)	0.39 (0.26)	0.36 (0.24)	0.88 (0.17)	0.80 (0.21)	0.82 (0.21)	0.85 (0.18)
13	BDX	5053.68	208574	0.45 (0.29)	0.58 (0.31)	0.52 (0.31)	0.46 (0.28)	0.76 (0.28)	0.62 (0.33)	0.69 (0.31)	0.76 (0.25)
14	ZWC	4550.20	22181	0.65 (0.34)	0.67 (0.33)	0.63 (0.34)	0.62 (0.33)	0.50 (0.40)	0.48 (0.40)	0.53 (0.39)	0.55 (0.39)
15	CAR	3932.36	66432	0.60 (0.32)	0.62 (0.32)	0.57 (0.32)	0.59 (0.32)	0.59 (0.35)	0.56 (0.37)	0.63 (0.33)	0.60 (0.34)
16	GTC	3773.78	787020	0.32 (0.25)	0.35 (0.28)	0.25 (0.17)	0.26 (0.19)	0.86 (0.20)	0.84 (0.24)	0.93 (0.08)	0.92 (0.10)
17	KTY	3668.03	155110	0.49 (0.30)	0.45 (0.28)	0.53 (0.30)	0.52 (0.31)	0.73 (0.29)	0.76 (0.26)	0.69 (0.29)	0.69 (0.30)
18	ORB	3363.62	101850	0.55 (0.31)	0.47 (0.29)	0.52 (0.29)	0.57 (0.31)	0.65 (0.33)	0.75 (0.27)	0.70 (0.28)	0.64 (0.32)
19	STP	2929.64	74227	0.54 (0.32)	0.50 (0.31)	0.52 (0.32)	0.50 (0.31)	0.66 (0.34)	0.70 (0.31)	0.68 (0.32)	0.70 (0.32)
20	ECH	2104.99	162464	0.48 (0.31)	0.54 (0.32)	0.40 (0.27)	0.45 (0.28)	0.72 (0.30)	0.65 (0.33)	0.81 (0.22)	0.77 (0.25)
	Total	259034.50	21010718	-							

Notes: The 20 WSE–listed companies are labeled by ticker symbols and reported in decreasing order of the market value (MV) at the end of 2016. WS—the whole sample period 2 January 2005–30 December 2016; S1–the pre-crisis sub-period 6 September 2005–31 May 2007; S2—the crisis sub-period on the WSE 1 June 2007–27 February 2009; S3—the post-crisis sub-period 2 March 2009–19 November 2010. Standard deviations are given in parentheses.

**Table 4:** Pearson correlation coefficients between daily market depth values calculated using the alternative indicators (1) and (2).

	PKN	PKO	PEO	BZW	ING	KGH	MBK	LPP	BHW	OPL
WS	−0.927	−0.920	−0.922	−0.923	−0.946	−0.913	−0.922	−0.950	−0.938	−0.929
S1	−0.928	−0.936	−0.929	−0.934	−0.956	−0.912	−0.940	−0.955	−0.950	−0.918
S2	−0.934	−0.941	−0.944	−0.922	−0.948	−0.945	−0.925	−0.949	−0.945	−0.925
S3	−0.947	−0.931	−0.942	−0.943	−0.946	−0.944	−0.945	−0.953	−0.948	−0.929
	MIL	SNS	BDX	ZWC	CAR	GTC	KTY	ORB	STP	ECH
WS	−0.935	−0.929	−0.940	−0.953	−0.950	−0.928	−0.944	−0.948	−0.946	−0.944
S1	−0.946	−0.944	−0.949	−0.956	−0.952	−0.937	−0.939	−0.955	−0.943	−0.949
S2	−0.941	−0.941	−0.948	−0.952	−0.947	−0.944	−0.950	−0.946	−0.947	−0.940
S3	−0.938	−0.931	−0.945	−0.948	−0.952	−0.937	−0.950	−0.949	−0.946	−0.948

Notes: Notation as in Table 3.

**Table 5:** Summarized results of the significance test for the difference between two means of daily Entropy-based Market Depth (EMD) values for the group of 20 WSE–listed equities.

	PKN	PKO	PEO	BZW	ING	KGH	MBK	LPP	BHW	OPL	No. of $H_{0}$
WS/S1	$H_{0}$	$H_{1}$	$H_{1}$	$H_{0}$	$H_{1}$	$H_{0}$	$H_{1}$	$H_{1}$	$H_{1}$	$H_{0}$	4
WS/S2	$H_{0}$	$H_{0}$	$H_{0}$	$H_{1}$	$H_{1}$	$H_{1}$	$H_{0}$	$H_{0}$	$H_{1}$	$H_{0}$	6
WS/S3	$H_{0}$	$H_{0}$	$H_{0}$	$H_{1}$	$H_{1}$	$H_{0}$	$H_{1}$	$H_{1}$	$H_{1}$	$H_{0}$	5
S2/S1	$H_{0}$	$H_{1}$	$H_{1}$	$H_{1}$	$H_{1}$	$H_{0}$	$H_{1}$	$H_{1}$	$H_{0}$	$H_{0}$	4
S2/S3	$H_{0}$	$H_{0}$	$H_{0}$	$H_{0}$	$H_{1}$	$H_{0}$	$H_{1}$	$H_{1}$	$H_{0}$	$H_{0}$	7
S1/S3	$H_{0}$	$H_{1}$	$H_{1}$	$H_{1}$	$H_{1}$	$H_{0}$	$H_{1}$	$H_{1}$	$H_{0}$	$H_{0}$	4
No. of $H_{0}$	6	3	3	2	0	5	1	1	3	6	30
	MIL	SNS	BDX	ZWC	CAR	GTC	KTY	ORB	STP	ECH	No. of $H_{0}$
WS/S1	$H_{1}$	$H_{1}$	$H_{1}$	$H_{0}$	$H_{0}$	$H_{0}$	$H_{0}$	$H_{1}$	$H_{0}$	$H_{1}$	5
WS/S2	$H_{1}$	$H_{1}$	$H_{1}$	$H_{0}$	$H_{0}$	$H_{1}$	$H_{0}$	$H_{1}$	$H_{0}$	$H_{1}$	4
WS/S3	$H_{1}$	$H_{0}$	$H_{0}$	$H_{0}$	$H_{0}$	$H_{1}$	$H_{0}$	$H_{0}$	$H_{0}$	$H_{1}$	7
S2/S1	$H_{0}$	$H_{0}$	$H_{0}$	$H_{0}$	$H_{0}$	$H_{1}$	$H_{1}$	$H_{0}$	$H_{0}$	$H_{1}$	7
S2/S3	$H_{1}$	$H_{0}$	$H_{1}$	$H_{0}$	$H_{0}$	$H_{0}$	$H_{0}$	$H_{0}$	$H_{0}$	$H_{0}$	8
S1/S3	$H_{1}$	$H_{1}$	$H_{1}$	$H_{0}$	$H_{0}$	$H_{1}$	$H_{1}$	$H_{1}$	$H_{0}$	$H_{1}$	3
No. of $H_{0}$	1	3	2	6	6	2	4	3	6	1	34

Notes: Notation as in Table 3. The critical value of Z-statistic at 0.25% significance level is equal to 3.03 for each test.

**Table 6:** Market trading schedule on the WSE equities–continuous trading system.

Market Phase	Time	Hours
Opening call	8:30 am–9:00 am	
Opening auction	9:00 am	
Continuous trading Closing call	9:00 am–4:50 pm 4:50 pm–5:00 pm	*H*_1_: 9:00 am–10:00 am *H*_2_: 10:00 am–11:00 am *H*_3_: 11:00 am–12:00 am *H*_4_: 12:00 am–1:00 pm *H*_5_: 1:00 pm–2:00 pm *H*_6_: 2:00 pm–3:00 pm *H*_7_: 3:00 pm–4:00 pm *H*_8_: 4:00 pm–5:00 pm
Closing auction	5:00 pm	
Trading at last	5:00 pm–5:05 pm	

Source: The WSE website (https://gpw.pl/session-details; accessed date 15 February 2021).

**Table 7:** Summarized findings of hourly patterns in the EMD indicator for the group of 20 WSE–listed equities.

	PKN	PKO	PEO	BZW	ING	KGH	MBK	LPP	BHW	OPL
WS	U-similar	U-similar	U-similar	U-similar	U-similar	U-similar	U-similar	U-similar	U-similar	U-similar
S1	M-similar	M-similar	M-similar	M-similar	M-similar	M-similar	M-similar	M-similar	M-similar	M-similar
S2	M-similar	M-similar	M-similar	M-similar	M-similar	M-similar	M-similar	Other	Other	M-similar
S3	U-similar	U-similar	U-similar	U-similar	U-similar	U-similar	U-similar	Other	U-similar	U-similar
	MIL	SNS	BDX	ZWC	CAR	GTC	KTY	ORB	STP	ECH
WS	U-similar	U-similar	U-similar	Other	U-similar	U-similar	M-similar	M-similar	U-similar	U-similar
S1	M-similar	M-similar	Other	Other	Other	U-similar	M-similar	M-similar	Other	M-similar
S2	M-similar	M-similar	M-similar	M-similar	U-similar	U-similar	M-similar	M-similar	M-similar	M-similar
S3	U-similar	U-similar	U-similar	Other	U-similar	U-similar	Other	U-similar	U-similar	U-similar

Notes: Notation as in Table 3. Based on Figure 2 and Figure A2, Figure A3 and Figure A4 (Appendix D).

**Figure A1:**
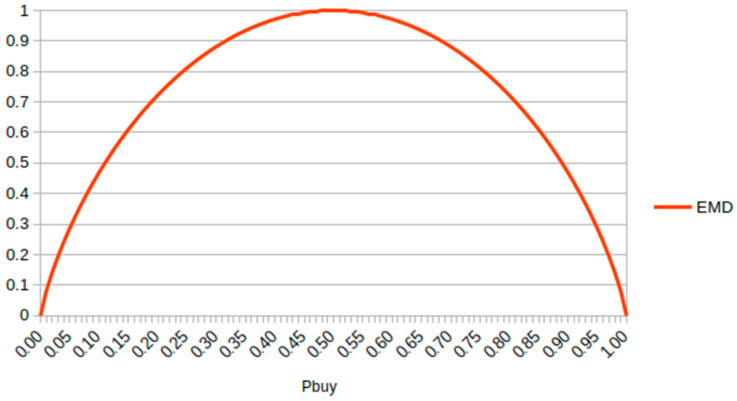
The Entropy-based Market Depth (EMD) as the function of probability $p=P_{buy}$.

## Data Availability

The data presented in this study are available on request from the corresponding author. The data are not publicly available at http://bossa.pl since 4 January 2021.

## Appendix A

Entropy is a measure that is used to summarize the information content of a probability distribution. Specifically, the Shannon information entropy quantifies the expected value of information contained in a discrete distribution [10]. The entropy in the case of two possibilities with probabilities p and 1−p can be represented as a function of p [1] (p. 394). Given the probabilities defined by Equations (3) and (4), we can set $P_{buy}=p$ and $P_{sell}=1-p$, and then the EMD indicator (2) can be directly written as the function of probability $p=P_{buy}$:

(A1) $$\begin{array}{l} EMD=\frac{-1}{\log(2)}\left(P_{buy}\cdot \log\left(P_{buy}\right)+P_{sell}\cdot \log\left(P_{sell}\right)\right)\\ \;=\frac{-1}{\log(2)}\left(p\log\left(p\right)+\left(1-p\right)\log\left(1-p\right)\right)=f\left(p\right) \end{array}$$

By analogy with the Shannon entropy, the f(p) function (A1) has several important properties which substantiate it as a reasonable measure of choice or information, and all of them are documented in [1]. In this paper, we focus on some basic properties, especially in the context of a binary distribution, and most of them are analyzed in Section 2.2. However, it is crucial to add that the f(p) function (A1) is non-negative, continuous and differentiable at each point in its domain. For instance, these properties allow us to assess the sensitivity of the EMD to changes in probability p as the argument of function f(p). The results could be interesting for practitioners as, from an investor’s point of view, it is important to know how do changes in probability $p=P_{buy}$ (connected with cumulated trading volume) affect the EMD value (it could be a possible direction for further investigation).

Figure A1 illustrates the plot of the EMD as the function of probability $p=P_{buy}$. It is important to notice that the EMD plot is identical with the plot of entropy presented in the Shannon’s seminal paper [1] (p. 394), and it confirms that the EMD measures both market liquidity and market entropy.

## Appendix B

Table A1 presents the Lee and Ready [45] algorithm inferring the initiator of a trade. The midpoint price $P_{t}^{mid}$ at time t is calculated as the arithmetic mean of the low price $P_{t}^{L}$ and the high price $P_{t}^{H}$ at time t, i.e., $P_{t}^{mid}=\frac{P_{t}^{L}+P_{t}^{H}}{2}$. The transaction price $P_{t}$ at time t is approximated by the closing price. The opening trade is treated as being unclassified according to the LR procedure.

**Table A1:** The Lee-Ready algorithm inferring the initiator of a trade.

Conditions		
**Stage I**		
$P_{t}>P_{t}^{mid}$	Trade is classified as buyer-initiated	
$P_{t}<P_{t}^{mid}$	Trade is classified as seller-initiated	
$P_{t}=P_{t}^{mid}$	Then:	
	**Stage II**	
	$P_{t}^{mid}>P_{t-1}$	Trade is classified as buyer-initiated
	$P_{t}^{mid}<P_{t-1}$	Trade is classified as seller-initiated
	$P_{t}^{mid}=P_{t-1}$	The decision is taken using the sign of the last non-zero price change $P_{t-k}$.
		$P_{t}>P_{t-k}$ Trade is classified as buyer-initiated
		$P_{t}<P_{t-k}$ Trade is classified as seller-initiated

Source: [56] (p. 6).

## Appendix C

Table A2 reports the number of trading days for each company and investigated period excluding the days when all of the transactions within a day are unclassified based on the Lee and Ready [45] algorithm inferring the initiator of a trade. These numbers are necessary to test the hypothesis (5) as the OR and EMD indicators are not defined when: (1) all transactions within an analyzed time period are unclassified, and (2) an analyzed time period is a zero-volume period, which means the total lack of transactions. Additionally, one can observe that for the most liquid companies (namely PKN, PKO, PEO, ING, KGH, MBK, BHW, OPL, MIL, SNS, GTC) the numbers of days reported in Table A2 are equal (or almost equal) to the particular sample size, respectively.

**Table A2:** The number of trading days excluding the days when: (1) all of the transactions within a day are unclassified, and (2) total daily trading volume is equal to zero.

	PKN	PKO	PEO	BZW	ING	KGH	MBK	LPP	BHW	OPL
WS	3005	3004	3005	2979	3004	3005	3005	2931	3004	3005
S1	436	436	436	436	436	436	436	411	436	436
S2	436	436	436	436	436	436	436	430	436	436
S3	436	436	436	436	436	436	436	424	436	436
	MIL	SNS	BDX	ZWC	CAR	GTC	KTY	ORB	STP	ECH
WS	3005	3005	2983	2433	2867	3003	2977	2962	2884	2967
S1	436	436	432	354	400	436	435	436	416	426
S2	436	436	435	385	433	436	436	436	432	436
S3	436	436	435	392	427	436	434	434	435	436

Notes: Notation as in Table 3. The sample size: WS (3005 trading days); S1, S2, S3 (436 trading days).

## Appendix D

Figure A2, Figure A3 and Figure A4 illustrate intra-day seasonality results within three sub-periods S1, S2, S3 for the whole group of 20 WSE–listed companies investigated in this study. Notation as in Table 3. Information about trading hours *H*_1_–*H*_8_ based on Table 6.
